# Human Papillomavirus L2 Capsid Protein Stabilizes γ-Secretase during Viral Infection

**DOI:** 10.3390/v14040804

**Published:** 2022-04-13

**Authors:** Mac Crite, Daniel DiMaio

**Affiliations:** 1 Department of Microbial Pathogenesis, Yale University, New Haven, CT 06511, USA; mac.crite@yale.edu; 2 Department of Genetics, Yale University, New Haven, CT 06511, USA

**Keywords:** papillomavirus, γ-secretase, viral entry, transmembrane, retrograde, retromer, endosome

## Abstract

Intracellular trafficking of human papillomavirus (HPV) during virus entry requires γ-secretase, a cellular protease consisting of a complex of four cellular transmembrane (TM) proteins. γ-secretase typically cleaves substrate proteins but it plays a non-canonical role during HPV entry. γ-secretase binds to the HPV minor capsid protein L2 and facilitates its insertion into the endosomal membrane. After insertion, L2 protrudes into the cytoplasm, which allows HPV to bind other cellular factors required for proper virus trafficking into the retrograde transport pathway. Here, we further characterize the interaction between γ-secretase and HPV L2. We show that γ-secretase is required for cytoplasmic protrusion of L2 and that L2 associates strongly with the PS1 catalytic subunit of γ-secretase and stabilizes the γ-secretase complex. Mutational studies revealed that a putative TM domain in HPV16 L2 cannot be replaced by a foreign TM domain, that infectivity of HPV TM mutants is tightly correlated with γ-secretase binding and stabilization, and that the L2 TM domain is required for protrusion of the L2 protein into the cytoplasm. These results provide new insight into the interaction between γ-secretase and L2 and highlight the importance of the native HPV L2 TM domain for proper virus trafficking during entry.

## 1. Introduction

Human papillomaviruses (HPV) are important pathogens that play an etiological role in approximately 5% of all cancers worldwide, including at least 99% of cervical cancers [1]. There are effective prophylactic vaccines against some HPV types, but these vaccines have no effect on ongoing HPV infection, do not protect against all HPV types, and are not widely deployed in much of the world. Therefore, study of HPV infection may lead to important new therapeutics to reduce the spread of the virus and disease burden. In addition, thorough investigation of HPV infection is likely to provide new insights into many aspects of cell biology and biochemistry [2].

The HPV virion is non-enveloped and contains 360 molecules of the major capsid protein, L1, and up to 72 molecules of the minor capsid protein, L2 [3]. Upon binding to heparan sulfate proteoglycans on the cell surface or extracellular matrix, HPV undergoes conformational changes and proteolytic cleavage and is transferred to an unidentified cell surface receptor for internalization [4,5]. HPV entry then occurs through the retrograde pathway: after cell internalization, the virus traffics from endosomes to the trans-Golgi network (TGN) and Golgi apparatus, possibly the endoplasmic reticulum (ER), and finally the nucleus. Internalized HPV remains within vesicular compartments until breakdown of the nuclear envelope during mitosis, which allows the viral genome to access cellular DNA replication machinery [6]. Virions assembled from L1 only are noninfectious and do not reach the nucleus, and many L2 mutants have been described that display trafficking defects [7,8,9,10,11,12,13]. These results show that L2 plays an important role in trafficking of the viral particle during HPV entry. Cellular proteins such as retromer and -secretase also play important roles in HPV entry.

A cell-penetrating peptide (CPP) in the C-terminus of L2 is also required for proper trafficking of HPV [9,14]. CPPs are short peptide segments that can transfer proteins and other cargo into cells [15]. Our results showed that the HPV L2 CPP mediates transfer of protein segments across intracellular membranes. Specifically, when internalized HPV is in the endosome, the CPP penetrates the endosomal membrane, and a segment of L2 upstream of the CPP protrudes through the endosomal membrane into the cytoplasm [9,16]. This allows L2 to bind to cytoplasmic entry factors such as the retromer complex, which normally mediates retrograde trafficking of cellular transmembrane (TM) cargo proteins from the endosome to TGN. Retromer-L2 binding is essential for HPV exiting the endosome and entering the retrograde transport pathway [8,17].

Early during entry when HPV resides in the endosome, HPV engages with γ-secretase, a protein complex comprised of four subunits (nicastrin [NCT], presenilin 1 [PS1], PEN2, and APH1), each of which have at least one TM domain [7,18]. Typically, γ-secretase recognizes and cleaves TM proteins such as Notch and the amyloid precursor protein (APP) within their TM domain [19]. Knockdown of any of the four γ-secretase subunits or treatment with chemical inhibitors of γ-secretase proteolytic activity, such as compound XXI, cause a severe defect in HPV infection and block intracellular HPV trafficking during entry [17,20,21,22]. Additionally, we showed that γ-secretase binds to L2 and promotes stable insertion of L2 into the endosome membrane [7]. The cellular protein p120 catenin is required for HPV-γ-secretase interaction [23]. The mechanistic details of how γ-secretase associates with L2 and promotes insertion of L2 into the membrane have not fully been elucidated. Dopachrome tautomerase (DCT), a γ-secretase substrate, supports HPV entry, but it is not known whether cleavage of DCT is required for HPV trafficking [24].

In vitro experiments with purified components showed that a short N-terminal segment of L2 appended to GFP binds directly to γ-secretase [7]. This segment of L2 contains a conserved hydrophobic, glycine-rich segment that appears to act as a TM domain, but it is less hydrophobic than canonical TM domains. Although γ-secretase can cleave within this putative TM domain, cleavage of L2 by γ-secretase is not required for successful infection. Glycine to valine mutations in this segment of HPV L2 inhibit the association of HPV with γ-secretase and retromer in infected cells, severely inhibit infection, and impair proper HPV trafficking to the TGN [7,10]. Others have also reported that some mutations in this TM segment of L2 drastically block HPV entry [10,11]. The ability of these mutants to associate with cellular factors such as γ-secretase was not investigated, and the precise nature of their trafficking defect was not determined. This segment of bovine papillomavirus (BPV) L2 is also important for infectivity and engages the SNARE protein syntaxin 18, but it is not known if syntaxin 18 is required for BPV or HPV infection [25,26].

Most of the information summarized above was obtained through studies of HPV pseudoviruses (PsVs). HPV PsVs are comprised of the L1 and L2 proteins, which assemble into a capsid that encapsidates a plasmid that expresses a reporter protein such as green fluorescent protein (GFP). The reporter gene is expressed upon successful infection of target cells and delivery of the reporter plasmid to the nucleus. PsVs provide several advantages for the study of HPV entry. They are easy to produce in monolayer cultures of cells, whereas the production of authentic virus requires stratified epithelial cells. Second, PsVs are non-pathogenic because they lack viral genes. Third, infectivity is simple to quantify, for example, by flow cytometry for reporter protein fluorescence. Finally, unlike the situation with authentic virus, it is relatively simple to generate and characterize specific viral mutants, including L2 proteins containing an epitope tag, L2 mutants that are defective for entry, and PsVs that lack L2. Although PsVs do not express any viral genes, they appear to act remarkably similarly to authentic virions during entry [27]. Thus, they are well suited to investigate the entry process, which is mediated solely by the viral capsid proteins in the incoming virus particle working in concert with cellular proteins.

In this paper, we use HPV PsVs to show that infection by divergent HPV types stabilizes the γ-secretase complex in multiple cell lines. γ-secretase stabilization requires the L2 protein and is tightly correlated with the infectivity of L2 mutant PsVs. Additionally, we found that γ-secretase activity and the HPV TM domain are required for protrusion of L2 into the cytoplasm. Taken together, our findings suggest that stabilization of γ-secretase by HPV may promote stable membrane protrusion of L2 and that specific residues within the putative TM domain of L2 play a critical role in this process.

## 2. Materials and Methods

### 2.1. Cell Lines

HeLa S3 cells were purchased from American Type Culture Collection (Manassas, VA, USA) HaCaT cells were purchased from AddexBio Technologies (San Diego, CA, USA), and 293TT cells were obtained from Christopher Buck (NIH). All cell lines were cultured at 37 °C and 5% CO_2_ in DMEM supplemented with 2% HEPES, 10% fetal bovine serum, l-glutamine, and 100 units/mL penicillin–streptomycin.

### 2.2. Generation of Mutant HPV16 Pseudovirus Packaging Plasmids

A silent unique AvrII restriction site was inserted downstream of the putative L2 TM domain in the p16SheLL-3X FLAG vector using the Q5 site-directed mutagenesis protocol and Phusion High Fidelity Polymerase. This plasmid was used to generate the putative TM domain mutants by inserting gBlocks (Integrated DNA Technologies, Coralville, IA, USA) containing the relevant mutation between the AvrII restriction site and an XbaI site upstream of the L2 start codon. The GV mutant was previously described [7]. For split GFP experiments, we generated PsVs from p16sheLL-CPP-GFP11, which contains seven copies of GFP11 appended to the C-terminus of L2 in wild-type or mutant PsV preparations [9].

### 2.3. HPV Pseudovirus Production

HPV PsVs were produced by using polyethylene imine (PEI) (MilliporeSigma, Burlington, MA, USA) to transfect 293TT cells with wild-type or mutant p16L1-GFP (to generate L1 only PsVs), p16SheLL, p16SheLL-3XFLAG, p5SheLL, or p16SheLL-GFP11 together with pCAG-HcRed or pCINeo-GFP as a reporter gene. PsV was collected and purified using an OptiPrep gradient (MilliporeSigma), as described previously [28]. In brief, cells were collected 72 h post transfection in siliconized tubes and lysed. Crude PsV preps were incubated overnight at 37 °C to allow capsid maturation. Matured PsV preps were loaded on an OptiPrep gradient and centrifuged at 50,000× *g* for 4 h at 4 °C in a SW-55Ti Beckman Ultracentrifuge rotor (Beckman Coulter, Indianapolis, IN, USA) rotor. Fractions were collected, and L1 and L2 levels of purified PsV preparations were assessed by SDS-PAGE followed by Coomassie blue staining or by immunoblotting with antibodies recognizing L1 and L2.

### 2.4. qPCR for Reporter Gene Quantitation

Encapsidated HcRed genomes were quantitated by qPCR as described previously [8]. In brief, gradient-purified PsV preps were treated with DNAse I to remove unencapsidated DNA, followed by proteinase K to digest capsids. The reporter plasmid was purified with the Qiagen DNeasy Blood and Tissue kit (Qiagen, Germantown, MD, USA) followed by qRT-PCR with primers specific to HcRed in comparison to a standard curve to determine viral genome quantity.

### 2.5. Infectivity

One day before infection, 1 × 10^5^ HeLa S3 or HaCaT cells were plated in 12-well plates. Cells were mock-infected or infected with wild-type or mutant PsV, and flow cytometry was used to measure reporter gene expression 48 h post infection (h.p.i.). The amount of mutant PsV used to infect cells was normalized to wild-type HPV16 PsV by using equal levels of L1 and L2 in purified PsV or qRT-PCR for the encapsidated reporter plasmid.

### 2.6. Co-Immunoprecipitation of L2-FLAG and γ-Secretase

Twenty-four hours before infection, 1 × 10^6^ HeLa S3 cells were plated in 6 cm dishes. Cells were infected with wild-type or mutant HPV PsV at a multiplicity of infection (MOI) of 50 for 16 h, unless otherwise indicated. Cells were scraped off the dishes and lysed in HN-DMNG lysis buffer (50 mM HEPES pH 7.5, 150 mM NaCl, 1% decyl maltose neopentyl glycol (DMNG) (Anatrace, Inc., Maumee, OH, USA) supplemented with HALT protease inhibitors) on ice for 45 min. For the co-immunoprecipitation experiment, in which γ-secretase subunits were stripped away from HPV using different amounts of detergents, samples were prepared the same, except HN lysis buffer with combinations of *N*-Dodecyl-B-d-Maltoside (DDM) (Anatrace) and NP40 was used (total detergent concentration, 1%). Cell debris was removed from the sample by centrifuging at 16.1× *g* for 15 min at 4 °C. The supernatant was incubated with anti-FLAG antibody for 4–6 h at 4 °C on a rotating tube rack. Then, 50 µL of Protein G magnetic beads (Thermo Fisher Scientific, Waltham, MA, USA) were washed in TBS-T, added to the lysate, and incubated at 4 °C overnight. Samples were washed three times with TBS-T and eluted from the beads using 2X Laemmli sample buffer (4% SDS, 20% glycerol, 0.004% bromophenol blue, 0.125 M Tris-Cl, pH 6.8, 10% Dithiothreitol (DTT)) at 100 °C. The entire sample was subjected to SDS-PAGE, transferred to a 0.2 µM PVDF membrane, blocked with 5% milk in TBS-T, and immunoblotted with antibodies recognizing the FLAG tag on the C-terminus of HPV16 L2 and γ-secretase substrates. See Table S2 for antibody information and dilutions.

### 2.7. Assay for γ-Secretase–HPV Association and γ-Secretase Stabilization

Twenty-four hours before infection, 1 × 10^6^ HeLa S3 cells were plated in 6 cm dishes. Cells were infected with wild-type or mutant HPV PsV at a MOI of 50 for 16 h. Cells were scraped off the dishes and lysed in HN-DDM lysis buffer (50 mM HEPES pH 7.5, 150 mM NaCl, 1% DDM supplemented with HALT protease inhibitors) on ice for 45 min. Where indicated, cells were lysed in HN lysis buffer containing 1% of the indicated detergent (CHAPSO, DDM, or NP40). Cell debris was removed from the sample by centrifuging at 16.1× *g* for 15 min at 4 °C. The supernatant was incubated with anti-PS1 or -APH1 antibody for 4–6 h at 4 °C on a rotating tube rack. Then, 50 µL of Protein G magnetic beads were washed, added to the lysate, and incubated at 4 °C overnight. Samples were washed three times and eluted from the beads using 2X Laemmli sample buffer at 100 °C. The entire sample was subjected to SDS-PAGE, transferred to a 0.2 µM PVDF membrane, blocked with 5% milk in TBS-T, and immunoblotted with antibodies recognizing HPV16 L1, the FLAG tag on the C-terminus of HPV16 L2 or γ-secretase substrates.

### 2.8. Split GFP Assay

HaCaT cells expressing GFP1-10NES were generated as described previously [9]. For the split GFP assay, 2.5 × 10^4^ GFP1-10NES-expressing cells were plated in eight-chambered glass slides overnight. Cells were treated with 1 µM of XXI γ-secretase inhibitor (MilliporeSigma) for 30 min prior to infection or left untreated, then infected with wild-type HPV16 PsV with a FLAG tag or with seven copies of GFP11 fused to the C-terminus of L2, or with mutant HPV PsV with seven copies of GFP11 fused to the C-terminus of L2. All PsVs were infected at a MOI of 2000. Live cells were stained 3 h post infection with Hoescht 33342 for 15 min at 37 °C to visualize DNA. Live cells were analyzed for reconstituted GFP fluorescence using a Leica SP5 confocal microscope.

## 3. Results

### 3.1. Characterization of the Interaction between γ-Secretase and HPV L2

We previously reported that the human papillomavirus (HPV) L2 minor capsid protein binds to γ-secretase during HPV entry [7]. In these studies, we used immunoprecipitation of infected cell extracts with an antibody recognizing a FLAG epitope tag on the HPV16 L2 protein in the PsV followed by mass spectrometry and identified peptides originating from all four subunits of γ-secretase. Co-immunoprecipitation (co-IP) was then used to confirm that L2 is in a complex with γ-secretase subunits PS1 and NCT during virus entry, but the presence of the other γ-secretase subunits in the L2 complex during infection was not assessed by co-IP. To further characterize the interaction between HPV and γ-secretase, we first performed co-IP in cells infected with HPV16 PsVs to confirm and extend our previous results. Unless stated otherwise, all infections in this paper used HPV16 PsV containing L2 with three tandem repeats of the FLAG tag at the C-terminus of the wild-type or mutant L2 protein.

**Figure 1:**
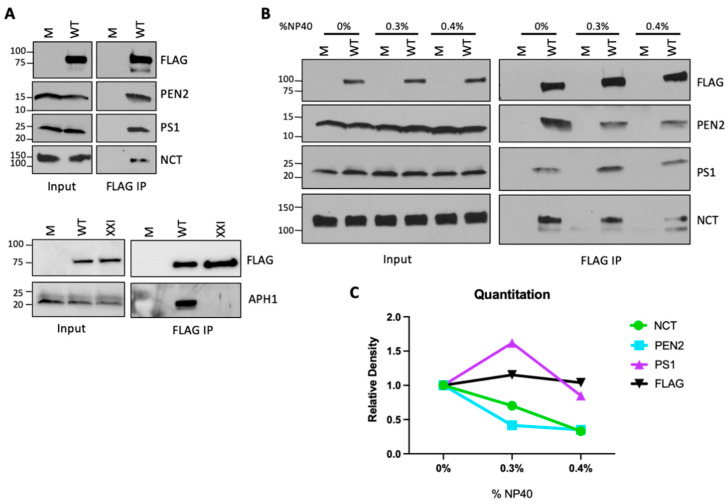
Characterizing the interaction between HPV and γ-secretase. (**A**) HeLa cells were treated with 1 µM XX1 γ-secretase inhibitor as indicated (bottom panels) or left untreated, then infected at MOI 50 with HPV16 PsV containing a 3XFLAG tag on the C-terminus of L2 (WT) or mock (M) infected. Cell lysates were collected 16 h.p.i. in lysis buffer containing 1% DMNG. L2 was immunoprecipitated with anti-FLAG, and samples were analyzed by Western blotting for HPV L2 (FLAG) and the indicated γ-secretase subunit. The inputs were total cell lysates without immunoprecipitation. Similar results were obtained in eight independent experiments. (**B**) HeLa cells were infected, immunoprecipitated, and analyzed as in (**A**), except the lysis buffer containing the indicated percentage of NP40 was supplemented with DDM to make the total detergent concentration 1%. Similar results were obtained in three independent experiments. (**C**) The ImageJ Gel Analysis tool was used to quantitate the relative density of each band in (**B**), normalized to the density of the respective band in samples lysed in 1% DDM (set to a relative density of 1).

HeLa cells were infected with HPV16 PsV, and at 16 h post infection (h.p.i.), extracts were prepared in buffer containing 1% decyl maltose neopentyl glycol (DMNG), a weak non-ionic detergent that solubilizes cell membranes but leaves most TM protein complexes intact. After immunoprecipitation with antibody recognizing FLAG, samples were subjected to SDS-PAGE and immunoblotting with antibodies recognizing γ-secretase subunits. As shown in Figure 1A (top panels), γ-secretase subunits PEN2, PS1, and nicastrin (NCT) are detected in the immunoprecipitates from infected but not uninfected cells, confirming that L2 is in a complex with all four subunits of γ-secretase. We also assessed binding of the γ-secretase subunit APH1 to L2 in cells in the presence and absence of a γ-secretase inhibitor, XXI. The anti-FLAG antibody immunoprecipitated APH1 in infected cells, showing that HPV is in a complex with APH1, as well as with the other γ-secretase subunits, and XXI blocked this association, as we reported earlier for PS1 [7]. These results show that HPV associates with all four γ-secretase subunits during infection.

To explore the interaction between L2 and γ-secretase in more detail, we next assessed the γ-secretase–HPV16 interaction in detergents of different stringencies. Published work suggested that γ-secretase can be disassociated into two subcomplexes of PS1–PEN2 and APH1–NCT under mild detergent conditions and into all four independent subunits under more stringent conditions [29]. In our experiments, infected cells were lysed in varying concentrations of a mild and a more stringent detergent to partially disassociate the L2–γ-secretase complex prior to immunoprecipitation and Western blotting. HeLa cells were mock-infected or infected with wild-type HPV16 PsV and lysed at 16 h.p.i. with buffer containing 1% *N*-Dodecyl-B-d-Maltoside (DDM) alone or with a buffer containing a mixture of different concentrations of NP40 and DDM. DDM, like DMNG, is a mild detergent that maintains most TM protein complexes, whereas NP40 is more stringent. Similar amounts of L2 and γ-secretase subunits were extracted in all conditions (Figure 1B and Figure S1A, left panels). The FLAG antibody immunoprecipitated similar amounts of L2 from infected cells at all stringencies (Figure 1B and Figure S1A, right panels). Additionally, similar amounts of PS1 were precipitated from all lysates, while levels of immunoprecipitated PEN2 and NCT decreased as detergent stringency increased (Figure 1B,C and Figure S1A). Thus, the PS1–HPV interaction is least affected by increasing detergent stringency, suggesting that PS1 binds most directly to the L2 protein.

### 3.2. HPV Infection Stabilizes the γ-Secretase Complex

**Figure 2:**
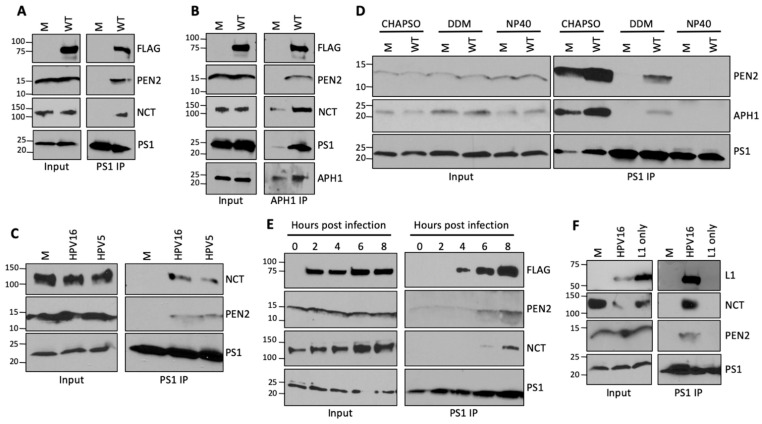
HPV L2 stabilizes the γ-secretase complex. (**A**) HeLa cells were infected as in Figure 1A, and lysates were collected in lysis buffer containing 1% DDM. The PS1 subunit of γ-secretase was immunoprecipitated with anti-PS1 and samples were analyzed by Western blotting for HPV and the indicated γ-secretase subunit as in Figure 1A. Similar results were obtained in three independent experiments. (**B**) As in (**A**), except anti-APH1 was used for immunoprecipitation. Similar results were obtained in seven independent experiments. (**C**) As in (**A**), except cells were infected with HPV16 (WT) or HPV5 PsV. Similar results were obtained in two independent experiments. (**D**) As in (**A**), except cells were lysed in buffer containing 1% of the indicated detergent. Similar results were obtained in two independent experiments. (**E**) As in (**A**), except cells were harvested at the indicated time after addition of PsV. (**F**) As in (**A**), except cells were infected with complete PsV (HPV16) or L1-only HPV16 PsV. Similar results were obtained in an additional independent experiment using anti-APH1.

To confirm the association between HPV and γ-secretase, we next performed the reciprocal co-IP experiment, i.e., immunoprecipitation with an antibody recognizing a γ-secretase subunit followed by blotting for the FLAG tag on L2. HeLa cells were mock-infected or infected with wild-type HPV16 PsV for 16 h, and the samples were lysed in 1% DDM. The PS1 subunit of γ-secretase was immunoprecipitated, and samples were analyzed by gel electrophoresis and Western blotting for L2 and the other γ-secretase subunits. In infected cells, L2 and the other subunits of γ-secretase were immunoprecipitated by anti-PS1, as expected, confirming the association between incoming HPV16 and γ-secretase (Figure 2A). Surprisingly, in uninfected cells, the other γ-secretase subunits were not co-IPed by the antibody recognizing PS1. This result suggests that HPV16 infection stabilizes the γ-secretase complex. A similar result was obtained when an antibody to another γ-secretase subunit, APH1, was used for immunoprecipitation: co-IP of the other γ-secretase subunits was greatly increased in cells infected with HPV (Figure 2B). Finally, stabilization of γ-secretase was also observed in HeLa cells infected with HPV5, a cutaneous β-papillomavirus that is evolutionarily divergent from the mucosal α-papillomavirus, HPV16 (Figure 2C).

HPV might induce the formation of the complex or alter the arrangement of γ-secretase subunits in a preexisting complex in such a way as to strengthen the interactions between the γ-secretase subunits. To characterize the γ-secretase complex in the presence and absence of HPV infection, we performed PS1 IPs in weak (CHAPSO), intermediate (DDM), and strong (NP40) detergents. In the presence of the mildest detergent, CHAPSO, anti-PS1 immunoprecipitated the other components of the γ-secretase complex in both infected and uninfected cells, showing that the complex exists in the absence of HPV infection (Figure 2D). In the presence of the intermediate-strength detergent DDM, anti-PSI immunoprecipitated the other components of the γ-secretase complex only in cells infected by HPV, as shown above. In the presence of the strong detergent, NP40, anti-PS1 did not immunoprecipitate the other components of the complex, regardless of HPV infection (Figure 2D). These results suggest that HPV alters the preexisting γ-secretase complex to produce a complex that is resistant to dissociation by the intermediate strength detergent.

We also examined the kinetics of HPV-induced stabilization of γ-secretase. In a time-course experiment with immunoprecipitation with anti-PS1, the L2–γ-secretase interaction was first observed at 4 h.p.i., followed shortly thereafter by stabilization at 6 h.p.i., which increased at 8 h.p.i. (Figure 2E). Next, we determined whether the stabilization occurred in a second cell line and depended on the amount of inputted HPV16 PsV. HaCaT keratinocytes were infected at different MOIs between 25 and 400 and then subjected to coIP analysis using the anti-PSI antibody. As shown in Figure S1B, increasing amounts of L2, PEN2, and NCT were co-immunoprecipitated as the MOI increased, indicating that stabilization of γ-secretase is directly proportional to the amount of HPV added to the cells. This dose-dependent effect was also observed in HeLa cells (data not shown). Taken together, these results suggest that γ-secretase stabilization directly results from an interaction with HPV.

To determine whether stabilization of γ-secretase required the L2 protein, HeLa cells were infected with HPV16 PsVs containing either both L1 and L2 (complete PsV) or only L1 (L1-only PsV) (Figure 2F and Figure S1C). At 16 h.p.i., cells were lysed in 1% DDM buffer and immunoprecipitated with a PS1 antibody. Samples were then subjected to gel electrophoresis and Western blot analysis by using an antibody that recognizes HPV L1 (because L2 was lacking from the L1-only PsV). Under these conditions, L1 was co-IPed with γ-secretase from cells infected with complete PsV containing L1 and L2, but did not co-IP from cells infected with L1-only PsV (Figure 2F), indicating that L2 is necessary for binding between HPV and γ-secretase in infected cells, consistent with our published in vitro binding experiments [7]. Importantly, the PS1 antibody immunoprecipitated the other components of the γ-secretase complex only from cells infected with complete PsV containing L1 and L2 and not from cells infected with L1-only PsV, suggesting that the L2 protein is also required for stabilization of γ-secretase.

### 3.3. Effect of Mutations within the L2 Transmembrane Domain

**Figure 3:**
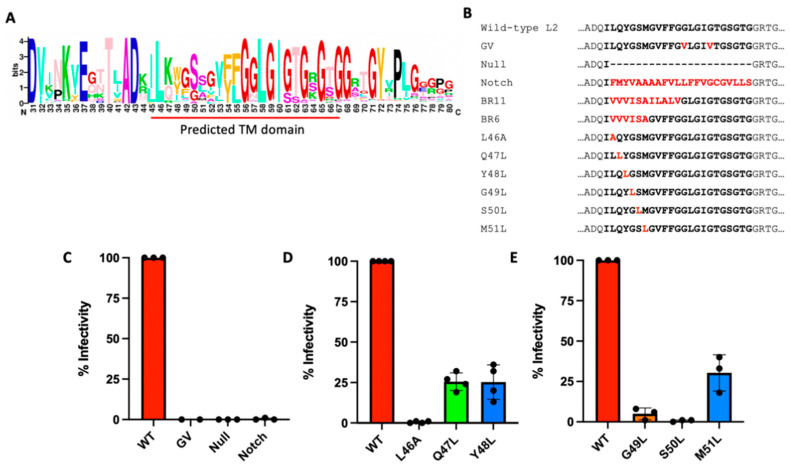
Mutations within the transmembrane domain of L2 affect infectivity. (**A**) Sequence logo of over 300 papillomavirus types showing the predicated TM domain (underlined in red) and the surrounding sequence, numbered according to the sequence of HPV16. (**B**) TM domain mutants. The top line shows the sequence of wild-type HPV16 L2 beginning at position 42. Mutations are highlighted in red and the TM domain is emboldened. (**C**–**E**) The indicated PsVs (normalized for packaged reporter plasmid genomes) were used to infect HeLa cells. Samples were analyzed 48 h.p.i. by flow cytometry for HcRed reporter gene expression. Graphs show percent infectivity, normalized to infectivity of cells infected with wild-type HPV16 PsV (set at 100%), from three or four experiments. Each dot represents a result from an independent experiment.

Some mutations within the putative N-terminal TM domain of L2 drastically inhibit infection [10,11]. Figure 3A shows a sequence logo of the TM domain and surrounding amino acids of over 300 different papillomaviruses. The C-terminal half of this TM domain contains multiple short glycine repeats (GxxxG) that have been proposed to mediate dimerization between L2 proteins [10]. We previously reported that a double mutation in the putative TM domain of L2 that changes glycine 57 and glycine 61 to valines (G57V/G61V, the GV mutant) blocks association with γ-secretase and prevents HPV from trafficking to the TGN and other distal retrograde compartments [7].

Here, we constructed and tested additional TM mutants of HPV L2 to determine the features of the TM domain that were required for infection (Figure 3B). All the mutants tested appear to assemble properly, based on the relative amounts of L1 and L2 in purified PsV preps and successful packaging of reporter plasmid DNA (Figure S2A–C, Table S1). Flow cytometry of reporter gene expression was used to measure infectivity after cells were infected with purified PsVs containing equal amounts of encapsidated reporter plasmid DNA. First, we constructed a mutant in which the L2 TM domain was completely removed (Null) and a mutant in which the TM domain was replaced with the TM domain from the Notch protein (Notch), a canonical γ-secretase substrate. Neither mutant was infectious (Figure 3C), indicating that at least a portion of the L2 TM domain was specifically required for infection and that this requirement could not be satisfied by the TM domain of a bona fide γ-secretase substrate protein.

We then constructed and tested two TM mutants where either the N-terminal six (BR6) or eleven (BR11) amino acids of the predicted L2 TM domain were replaced with those from a bona fide transmembrane protein, the platelet-derived growth factor receptor (PDGFR). Both of these mutants were also noninfectious, suggesting that specific residues within the first six amino acids of the L2 TM domain are required for infection (Figure S2G,H). We then constructed a series of point mutations in which we individually substituted the leucine at the first predicted TM position with alanine (L46A) or each of the next five amino acids of the putative TM domain with the hydrophobic amino acid leucine (Q47L, Y48L, G49L, S50L, and M51L). PsVs containing these mutations displayed variable levels of infectivity, with the L46A, G49L, and S50L mutants being noninfectious and the Q47L, Y48L, and M51L mutants infecting at roughly 25% of wild-type HPV (Figure 3D,E). Taken together, these experiments show that the wild-type TM domain of L2 is required for infection and that replacing it with the TM domain from a γ-secretase substrate will not rescue infection. Additionally, several individual amino acids within the first six residues of the TM domain are required for efficient infection.

### 3.4. Correlation between Infectivity, γ-Secretase Binding, and γ-Secretase Stabilization for Transmembrane Domain Mutants

**Figure 4:**
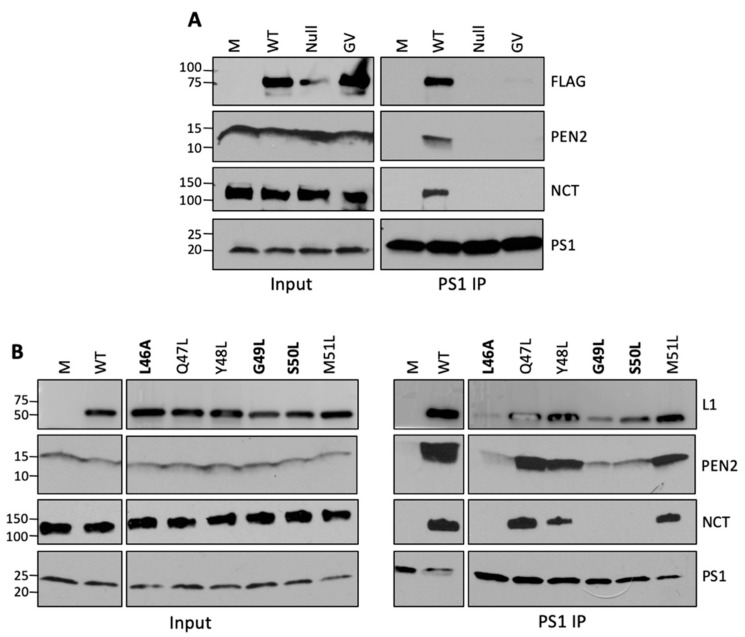
Infectious transmembrane mutants bind to and stabilize the γ-secretase complex. (**A**) As in Figure 2A, except cells were infected with the indicated wild-type and mutant PsVs normalized for the amounts of encapsidated genomes. Similar results were obtained in two additional independent experiments using anti-APH1. (**B**) As in panel (**A**). The most defective mutants are shown in bold font. All samples were analyzed on the same SDS-PAGE gel; an extraneous lane was removed between WT and the L46A mutant.

Next, we determined whether mutations in the L2 TM domain that inhibited infection also disrupted the interaction with γ-secretase. As a control, we analyzed the GV mutant, which does not bind γ-secretase and is non-infectious. To determine whether the GV and Null mutants bound and stabilized γ-secretase, cells were mock-infected or infected with wild-type, Null, or GV HPV16 PsV for 16 h, then lysed in 1% DDM, immuno-precipitated with antibody-recognizing PS1, and immunoblotted for L2 or the other γ-secretase subunits. PS1 associated with the wild-type but not with the mutant L2 protein PsVs (Figure 4A, top panel), showing that the putative TM domain of L2 is necessary for γ-secretase binding. Similarly, anti-PS1 immunoprecipitated NCT and PEN2 in cells infected with wild-type PsV but not with the GV or Null mutants (Figure 4A, middle panels). This result shows that the TM domain of L2 is also necessary for γ-secretase stabilization.

We also used the co-IP approach described above to determine which of the TM point mutations affected γ-secretase binding and stabilization. As shown in Figure 4B, there was a correlation between infectivity and the ability of the mutants to bind and stabilize γ-secretase: the three mutants that retained infectivity (albeit at a reduced level) bound to and stabilized the γ-secretase complex, whereas the three noninfectious mutants showed reduced binding to and stabilization of the complex. This suggests that binding of the TM domain of L2 to γ-secretase is important for infection and stabilization of γ-secretase.

### 3.5. γ-Secretase Activity and the Transmembrane Domain of L2 Are Required for Membrane Protrusion

**Figure 5:**
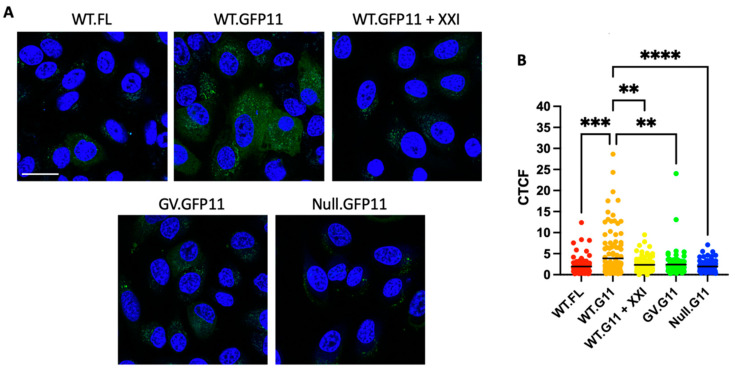
γ-secretase activity and the L2 transmembrane domain are required for membrane protrusion of L2. (**A**) Clonal HaCaT GFP1-10NES cells were treated with 1 µM XXI γ-secretase inhibitor for 30 min before infection or left untreated. Cells were then infected at MOI 2000 with wild-type HPV16 PsV containing a FLAG tag on the C-terminus of L2 (negative control) or the wild-type or mutant PsV containing a GFP11 tag. Three h.p.i., samples were stained with Hoechst 33342 to visualize nuclei (blue) and analyzed on a Leica SP5 confocal microscope for reconstituted GFP fluorescence (green). Scale bar represents approximately 25 µm. (**B**) Quantitated corrected total cellular fluorescence (CTCF = Integrated Density—(Area of selected cell X mean fluorescence of background)) of cells, as in (**A**), for ~300 cells per condition. A one-way ANOVA was used to assess the statistical significance of the results. ** *p* < 0.01; *** *p* < 0.001; **** *p* < 0.0001.

After HPV is internalized and enters the endosome, the L2 protein inserts into the endosome membrane and protrudes through it into the cytoplasm in order to bind retromer and be sorted into the retrograde transport pathway. γ-secretase acts as a chaperone to help L2 insert into the membrane. Here, we used a split GFP assay to test whether the L2 TM domain and γ-secretase activity are also required for membrane protrusion of L2. In this assay, seven copies of GFP11 were appended to the C-terminus of L2, which was used to generate PsV particles containing L1 and GFP11-tagged L2. GFP1-10 is expressed in the cytoplasm of HaCaT cells. Neither of these two GFP segments fluoresce on their own, but if the C-terminus of L2 protrudes through the endosome membrane during infection in cells expressing GFP1-10, GFP11 associates with GFP1-10 and reconstitutes cytoplasmic GFP fluorescence, which can be observed using confocal microscopy. HaCaT cells expressing cytoplasmic GFP1-10 were left untreated or treated with compound XXI, an inhibitor of γ-secretase. Cells were then infected with wild-type HPV16 PsV with or without GFP11 fused to L2 and analyzed by confocal microscopy at 3 h.p.i. When infected with wild-type HPV PsV lacking GFP11, infected GFP1-10 cells did not fluoresce (Figure 5A). However, if these cells were infected with GFP11-tagged HPV PsV, reconstituted cytoplasmic fluorescence was observed, as previously reported (Figure 5A; [9]). In contrast, when the cells were treated with XXI prior to infection, GFP11-tagged PsV did not generate reconstituted fluorescence, indicating that L2 is unable to protrude through the membrane in the absence of γ-secretase activity, consistent with our previous finding that γ-secretase plays a role in membrane insertion [7].

We also used the split GFP assay to test the ability of noninfectious L2 TM domain mutants to protrude into the cytoplasm. As shown in Figure 5A, GFP11-tagged GV and Null TM mutants failed to produce reconstituted fluorescence upon infection. Taken together with the inhibitor studies described above, these results indicate that the interaction of the L2 TM domain with γ-secretase is necessary for membrane protrusion of L2.

## 4. Discussion

Our study of the role of γ-secretase in HPV entry generated several interesting findings. First, HPV is in a complex with all four subunits of the γ-secretase complex and interacts with the catalytic subunit PS1. This result is consistent with recently published crystal structures of other substrates bound to γ-secretase, which showed that the TM helix of Notch and the amyloid precursor protein (APP) are surrounded by TM helices of PS1 [30,31]. The interaction of HPV with the catalytic subunit of γ-secretase appears vital for HPV entry, consistent with the fact that inhibitors of γ-secretase activity block HPV infection, even though protease activity is not required for successful infection.

Second, co-IP experiments with antibodies recognizing two different γ-secretase subunits showed that infection with HPV capsids containing L2 stabilizes the γ-secretase complex, whereas PsV capsids devoid of L2 do not stabilize the complex. Both α- and β-papillomaviruses stabilize γ-secretase, stabilization occurs at multiple MOIs in a dose-dependent manner, and stabilization occurs in HeLa cervical cancer cells and HaCaT immortalized keratinocytes. Thus, the ability of HPV to stabilize γ-secretase is conserved among diverse papillomavirus types in relevant cell types.

Third, we confirmed that the putative TM domain in the N-terminal portion of L2 is required for infection and γ-secretase binding. In addition, we showed that this segment of L2 is required for γ-secretase stabilization and for protrusion of L2 through the endosome membrane. By using the PsV system to generate and test TM domain mutants, we showed that the L2 TM domain could not be removed or replaced with the TM domain of a known γ-secretase substrate, and that single residue substitution mutations within the TM domain can have a large effect on HPV infection, HPV–γ-secretase interaction, and HPV trafficking. There is a correlation between γ-secretase stabilization, γ-secretase binding to HPV L2, and infectivity: the mutants that were the least infectious failed to bind to or stabilize the γ-secretase complex, while those that were partially infectious both bound to and stabilized γ-secretase. We did not identify a mutant that could bind to γ-secretase without stabilizing it. These results show that the intact L2 TM domain is vital for recognition of L2 by γ-secretase and imply that γ-secretase–L2 binding is required for γ-secretase stabilization and for the subsequent membrane protrusion and trafficking events. They further raise the possibility that γ-secretase stabilization plays a role in infection.

Our results extend the findings of others that point mutations in this portion of the L2 TM domain can inhibit infection by HPV16 and BPV PsVs [11,25]. The Jung group found that an L46K mutant HPV16 PsV was almost as infectious as wild-type PsV, whereas PsV containing a triple mutation of L46, Q47, and Y48 to three alanines was essentially noninfectious. Additionally, they showed that a replacement of residues 49–53 with five alanines retained roughly 90% infectivity of wild-type HPV PsV. Residues other than L46 within this six-amino acid region were not tested individually. We found that L46, G49, and S50 are critical for infection, and that mutations at Q47, Y48, and M51 impaired but did not abrogate infection. There are two key differences between the two studies that could contribute to these conflicting results. The Jung group assessed infectivity of their mutants in CHO-K1 hamster kidney cells, which is not a natural target for papillomavirus infection, whereas we assessed mutants in cells representative of the natural target of HPV. In addition, the Jung group replaced the residues with alanine, and we replaced them with leucine (the most abundant residue within TM domains [32]). In addition to the importance of individual residues, the sequence context of the L2 M domain also appears important. Mutations in the HPV16 L2 TM domain that replace the native residue with a residue found in other papillomavirus types, such as the G49L mutant, are much less infectious than the wild-type HPV16 PsV, even though leucine is present at this position in other HPV types, such as HPV95. Perhaps glycine but not leucine at this position in the context of the HPV16 TM domain allows for proper γ-secretase association, whereas leucine at this position in HPV95 allows association in the presence of the other sequence differences in the HPV95 TM domain.

The stabilization of γ-secretase by HPV L2 is intriguing. To our knowledge, stabilization of γ-secretase by substrates has not been previously reported, but some γ-secretase inhibitors have been reported to stabilize the γ-secretase complex by increasing interactions between γ-secretase subunits [33]. The structure of the γ-secretase complex bound to Notch or APP provides insight into substrate recognition [30,31]. Many of the residues in PS1 that contact Notch and APP differ, but there are some common elements. Both substrates interact with multiple TM domains of PS1 and induce the formation of two β-sheets in PS1 that interact with a β-sheet in the substrate, and hydrogen bonds involving S169 or G384 of PS1 anchor the interaction with the substrate TM domain. These common features may be important for substrate cleavage. While the structure of HPV L2 bound to γ-secretase is unknown, L2 likely interacts with PS1 in a different way than either canonical substrate, because L2 cleavage is minimal, the L2 TM domain scores lower than confirmed TM domains in TM prediction programs, and the TM domain of a known γ-secretase substrate cannot functionally substitute for the L2 TM domain [7,10]. We speculate that the interactions between HPV L2 and γ-secretase produce different structural arrangements of PS1 that stabilize its interactions with the other γ-secretase subunits. Alternatively, L2 itself may act as a “glue” by simultaneously contacting multiple γ-secretase subunits. It will be interesting to identify other cellular proteins that stabilize γ-secretase and to determine whether their TM domains can replace the L2 TM domain and support HPV infection.

Finally, protrusion of L2 into the cytoplasm requires γ-secretase activity, consistent with our previous finding that L2 membrane insertion requires γ-secretase. This suggests that the infectivity defect caused by γ-secretase inhibition is ultimately due to impaired L2 protrusion. The TM domain of L2 is also important for protrusion of L2 into the cytoplasm, presumably at least in part because it binds γ-secretase. The non-canonical TM sequence may be beneficial to the virus. As the L2 TM domain is less hydrophobic than typical TM domains, L2 might be able to move into and out of the membrane as necessary for proper viral trafficking. L2 protrudes through the membrane to bind γ-secretase and retromer, but perhaps it is released from membrane association in order to traffic to distal retrograde compartments. Experiments are underway to test these possibilities.

Overall, we have characterized the interaction between γ-secretase and HPV L2 and discovered that HPV infection stabilizes the γ-secretase complex, identified specific residues in the L2 TM domain that are required for stabilization, and provided evidence that stabilization may be important for infection. Additionally, γ-secretase activity and the L2 TM domain are required for L2 protrusion into the cytoplasm, a vital step in infection that allows L2 to bind to cytoplasmic cellular trafficking factors. γ-secretase stabilization may also be important for the processing, trafficking, and modulation of cellular γ-secretase substrates, and it will be interesting to investigate whether stabilization contributes to the pathogenesis of disorders related to improper γ-secretase function.

## Data Availability

All data needed to evaluate the conclusions in the paper are present in the paper and/or the Supplementary Materials. Additional data related to this paper may be requested from the authors.

## Supplementary Materials

The following supporting information can be downloaded at: https://www.mdpi.com/article/10.3390/v14040804/s1, Table S1: qRT-PCR for packaged reporter genomes; Table S2: List of antibodies used in this study; Figure S1: Related to Figure 1 and Figure 2; Figure S2: Related to Figure 3.
