# Supplementary material for: Human Papillomavirus L2 Capsid Protein Stabilizes γ-Secretase during Viral Infection
Source: Viruses. 2022 Apr 13;14(4):804. doi: 10.3390/v14040804 (PMC9027364; doi:10.3390/v14040804)
Supplement: Supplementary file 1 [file viruses-14-00804-s001.zip › viruses-1649186-supplementary.pdf]

viruses

MDPI

Supplementary Materials

Human Papillomavirus L2 Capsid Protein Stabilizes γ-Secretase during Viral Infection

Table S1. qRT-PCR for packaged reporter genomes.

Virus	Genomes/µL
WT HPV16	7.11E+05
Null	1.08E+06
Notch	2.79E+05
WT HPV16	3.73E+07
BR6	2.00E+06
BR11	1.85E+07
WT HPV16	2.14E+07
L46A	1.01E+07
Q47L	2.43E+07
Y48L	1.49E+07
WT HPV16	1.28E+06
G49L	1.79E+06
S50L	2.54E+06
M51L	5.75E+06

Table S2. List of antibodies used in this study.

Antigen	Source	Catalogue Number	Dilution	Application
FLAG M2	Sigma	F3165	1:5000	IP
FLAG HRP	Sigma	A8592	1:1000	WB
HPV16 L1	BD Biosciences	554171	1:1000	WB
PS1	Cell Signaling	5643S	1:1000	WB
			1:500	IP
PEN2	Cell Signaling	8598	1:500	WB
APH1	ThermoFisher	PA1-2010	1:1000	WB
			1:500	IP
NCT	Santa Cruz	Sc-376513	1:1000	WB

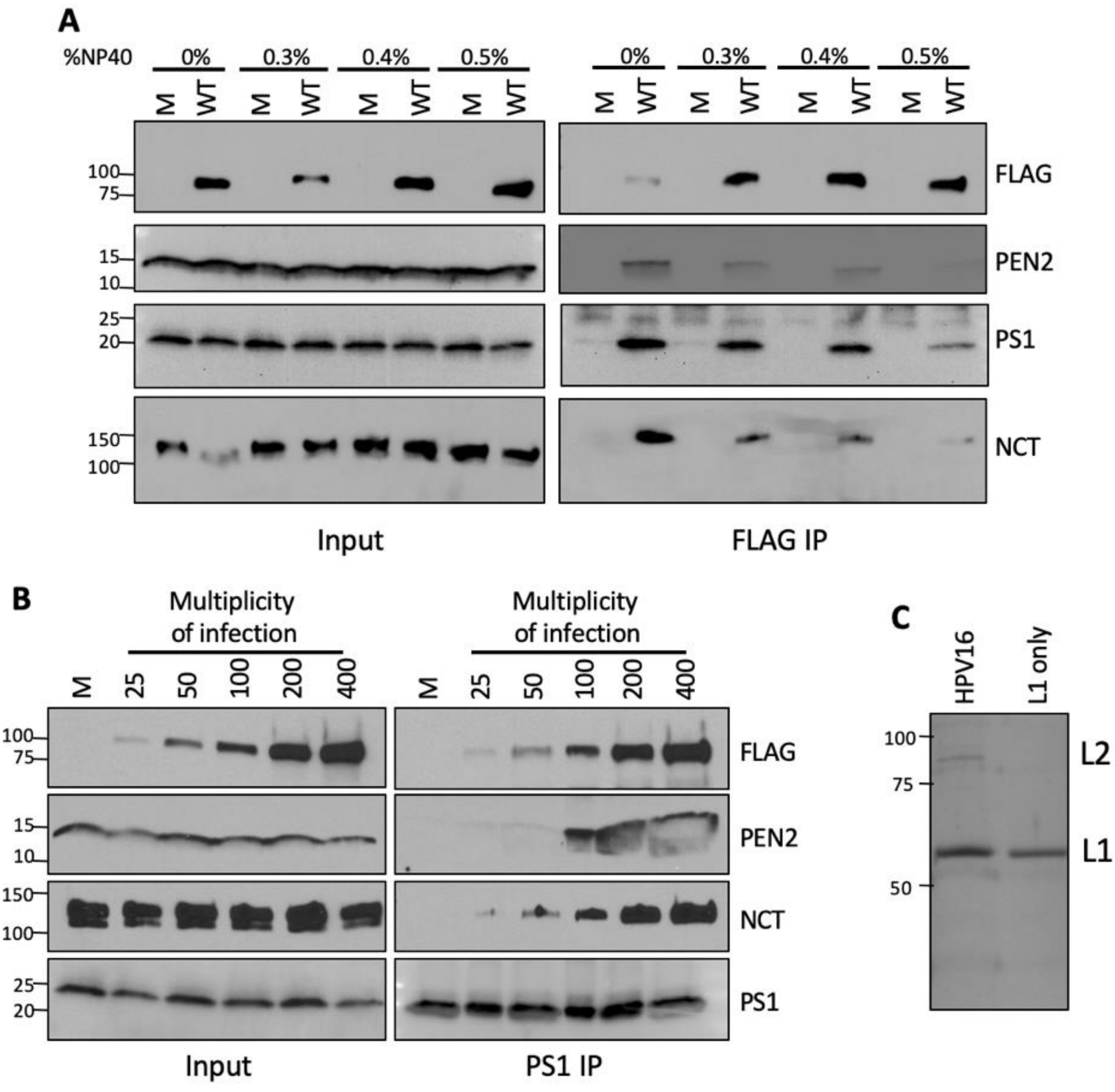

Figure S1. Related to figure 1 and 2.

(**A**) As in Figure 1B, with the lysis buffers containing the indicated amount of NP40. (**B**) As in Figure 2A, except HaCaT cells were infected at the indicated MOI (calculated in HeLa cells). (**C**) Complete PsV (HPV16) and L1-only PsV (normalized for packaged reporter plasmid DNA) were analyzed by SDS-PAGE followed by staining with Coomassie Brilliant Blue.

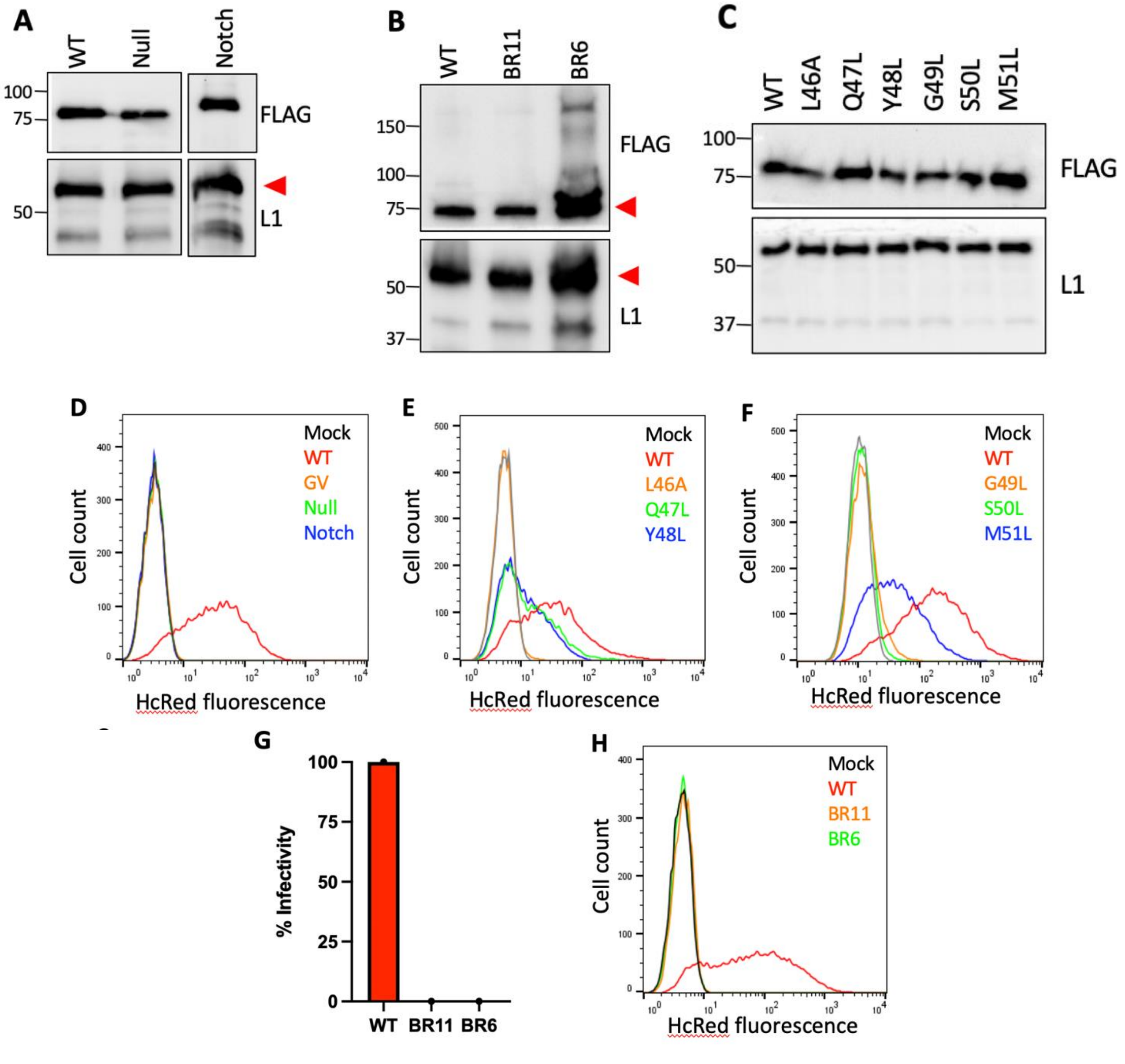

Figure S2. Related to figure 3.

(**A**) Normalized amounts of gradient-purified wild-type, L2 TM Null, and L2 TM Notch PsVs were analyzed by SDS-PAGE followed by western blotting for L1 or the FLAG tag on the C-terminus of HPV L2. Red arrowhead shows position of L1. All samples were analyzed on the same SDS-PAGE gel; an extraneous lane was removed between the Null and Notch mutants. (**B**) As in (**A**), except with the indicated TM mutants. Red arrowhead shows position of L1 and FLAG-tagged L2. (**C**) As in (**A**), except with the indicated TM point mutants. (**D-F**) Flow cytometry histograms of HcRed reporter gene expression used to generate the graphs shown in Figure 3C, 3D, and 3E, respectively. (**G**) Infection was analyzed as in Figure 3C for the indicated PsV. (**H**) Flow cytometry histograms are shown as in panel D-F.
